# Evaluation of thermal sensitivity is of potential clinical utility for the predictive, preventive, and personalized approach advancing metabolic syndrome management

**DOI:** 10.1007/s13167-022-00273-6

**Published:** 2022-02-18

**Authors:** Sujeong Mun, Kihyun Park, Siwoo Lee

**Affiliations:** KM Data Division, Korea Institute of Oriental Medicine, Daejeon, Republic of Korea

**Keywords:** Metabolic syndrome, Thermal sensitivity, Thermal intolerance, Thermal sensation, Predictive preventive personalized medicine (PPPM/3PM), Body mass index

## Abstract

A possible association between metabolic disorders and ambient temperature has been suggested, and cold exposure as a way of increasing energy expenditure has gained considerable interest for preventative/therapeutic measures toward metabolic disorders. Although thermal sensitivity, which has recently been studied in regard to its utility as a risk assessment/patient stratification for various diseases, might influence physiological responses to ambient temperature on an individual basis, more studies are needed. We aimed to investigate the association between self-identified thermal intolerance/sensation and metabolic syndrome (MetS) to verify the working hypothesis that individuals with altered thermal sensitivity may have a predisposition to MetS. We fitted generalized additive models for thermal intolerance/sensation using body mass index (BMI) and waist–hip ratio in women, and identified those with higher/lower thermal intolerance/sensation than those predicted by the models. Higher heat intolerance, higher heat sensation, and lower cold intolerance were associated with a higher prevalence of MetS. The risk of having MetS was increased in those who had two or three associated conditions compared with those with none of these conditions. In an analysis for MetS components, significant associations of thermal sensitivity were present with high glucose, triglyceride, and blood pressure levels. Overall, higher heat intolerance/sensation and lower cold intolerance were associated with increased prevalence of MetS even at a similar level of obesity. Our study indicates that evaluation of thermal sensitivity may help identify individuals at high risk for MetS, and lead to more advanced patient stratification and personalized treatment strategies for MetS, including cold-induced thermogenesis.

**Supplementary Information:**

The online version contains supplementary material available at 10.1007/s13167-022-00273-6.

## Introduction

Metabolic syndrome (MetS) refers to the clustering of inter-related metabolic disorders, including central obesity, hypertension, and increased glucose and abnormal cholesterol or triglyceride levels. Although the prevalence of MetS differs slightly according to the definitions of health care organizations, approximately 20–30% of the adults in most countries worldwide are reported to have MetS [1]. The presence of MetS has been reported to increase the risk of cardiovascular disease threefold; type II diabetes fivefold; and a plethora of cancers including breast, liver, pancreatic, and colon cancer [2]. Obesity, physical inactivity, and an atherogenic diet are widely recognized to increase the risk of developing MetS [3].

Recent studies have suggested a possible association between MetS and ambient temperature. Constantly comfortable environmental temperature in modernized societies might have resulted in the increased incidence of obesity and related metabolic disorders, including MetS and type II diabetes, by negatively affecting energy expenditure [4–7]. In this regard, excursions outside the thermal comfort zone, particularly prolonged cold exposure, have been suggested as a way of increasing energy expenditure to influence obesity, insulin sensitivity, and the immune system, which may be mediated via changes in brown adipose tissue (BAT) and metabolic pathways in skeletal muscles [8, 9].

Most observational studies on this issue have focused on the ambient temperature and its impact on metabolic diseases at the population level [4–7], while interventional studies have focused on the effect of a fixed cold acclimation protocol on metabolism [10–12]. However, the width and positioning of the thermoneutral zone largely vary in each individual, and the temperature that effectively activates BAT is also remarkably different between individuals [13]. Accordingly, it is conceivable that different thermal sensitivity of each individual might result in different metabolism even under the same environmental conditions. This means that studies are needed to investigate the association between thermal sensitivity at the individual level and metabolic diseases. Furthermore, the development of personalized protocols is eventually needed for the use of cold-induced thermogenesis (CIT) as a targeted preventive and therapeutic strategy for metabolic disorders.

The symptoms related to thermal sensitivity, such as feeling inappropriately cold/hot and cold hypersensitivity in hands/feet, have been generally considered as indicative of certain diseases including thyroid disorders, perimenopausal syndrome, or Raynaud’s phenomenon [14–17]. However, recent studies have focused on the utility of thermal sensitivity for risk assessment and patient stratification in a broader scope of diseases, such as ocular diseases, breast cancer, multiple sclerosis, MetS, and functional dyspepsia [18–24]. For example, one study demonstrated that feeling inadequately cold and cold extremities were prevalent in metastatic breast cancer patients and increased the risk of developing systemic hypoxic premetastatic niches long before breast malignancy is clinically manifested [21]. In regard to metabolic diseases, people with increased cold sensation in the extremities had a low incidence of MetS and related diseases, namely diabetes and dyslipidemia, even after adjustments for obesity levels [23, 24].

Obesity levels are closely related to thermal sensitivity. Obese individuals tend to feel hotter and have a higher skin temperature in their hands than the non-obese under the same environmental conditions [25–27]. Cold hypersensitivity in the hands/feet has been consistently reported to be more common in individuals with a lower body mass index (BMI) [28–30]. Flammer syndrome, which is characterized by increased cold sensation in the extremities and increased sensitivity to various stimuli, is also more frequently observed in lean individuals (anorexic phenotype) [31]. Meanwhile, obesity is also a powerful predictor of the key components of MetS [32, 33]. The obese phenotype, which has been presented as a counterpart concept of anorexic phenotype, confers an increased risk of various metabolic diseases including dyslipidemia, arteriosclerosis, and hypertension [31, 34]. Because obesity levels are significantly related to both thermal sensitivity and MetS, the independent association of thermal sensitivity and MetS should be investigated in a way that controls for obesity levels.

By defining the thermoneutral zone in each individual, thermal sensitivity could be evaluated objectively without the interference of subjective judgment; however, it is practically challenging, complex, and costly to define the thermoneutral zone of each individual. Each participant needs to be exposed to a specific temperature for several hours until thermal balance is achieved, and this should be repeated until both the lower and upper critical temperatures are manifested [35]*.* In addition, the thermoneutral zone varies according to seasonal changes due to the effect of acclimatization [36]. Nevertheless, thermal intolerance/sensation can be more conveniently and cost-effectively evaluated using a questionnaire, and self-identified cold intolerance/sensation could be a good index to predict physiological responses to cold exposure [37, 38].

## Working hypothesis

In the current study, we hypothesized that individuals with altered thermal sensitivity may have a predisposition to MetS and its components; thus, the evaluation of thermal sensitivity may help identify individuals at high risk for MetS and lead to more advanced patient stratification and personalized treatment strategies for MetS, including CIT. To verify our hypothesis, we first fitted a model to predict thermal intolerance/sensation based on obesity level to identify individuals with higher or lower thermal intolerance/sensation than the predicted values, which is a novel approach to evaluate thermal sensitivity based on obesity levels. We then investigated the independent association between thermal intolerance/sensation and MetS and its components.

## Methods

### Participants

This study analyzed the data from the Korean Medicine Daejeon Citizen Cohort (KDCC) study. The KDCC study based in Daejeon City, South Korea, used stratified cluster sampling and included adults who were aged 30–55 years without cancer or cardiovascular diseases (myocardial infarction, angina, and stroke/apoplexy). It included a health interview survey, physical examination, and laboratory tests [39]. Participants were recruited between June 2017 and December 2019, and baseline visit data were utilized in this study.

We only included women in the analysis because they are more sensitive to thermal changes [40, 41]. We excluded women at menopause, which might have affected thermal intolerance, and those with missing data on menopausal status. We also excluded those taking medications that might affect thermal intolerance, including oral contraceptives, psychiatric medications, antihistamines, and any medications for perimenopause and thyroid disorders. Additionally, we excluded women who had both strong cold and heat intolerance or sensation (≥ 8) and those who had neither cold nor heat intolerance/sensation (≤ 4). A total of 849 women were included in the analysis.

### Physical examination and laboratory tests

Height and body weight were measured using a digital stadiometer (BSM370, InBody, Seoul, South Korea) while the participant was standing barefoot. BMI was calculated as follows: weight (kg)/[height (m)]^2^. Waist circumference (WC) was measured at the navel level, and hip circumference was measured at the widest part of the gluteal region using a tape (Hoechstmass–Rollvix, Germany). Body composition was measured using a multi-frequency bioelectrical impedance analysis (Inbody770, InBody). Blood pressure (BP) was measured using an automatic blood pressure cuff (FT- 500R PLUS, Jawon Medical, South Korea).

Venous blood samples were collected in the morning following an overnight fast. Thirty minutes after collection, the sample was centrifuged for 10 min at 3450 rpm, and all samples were transported to the Seoul Clinical Laboratories (Seoul, South Korea) within 24 h. The homeostasis model assessment of insulin resistance (HOMA-IR) was calculated as follows: fasting blood glucose (FBG) (mg/dL) × fasting insulin (mIU/L)/405.

### Evaluation of thermal intolerance and sensation

The survey for thermal intolerance and sensation comprised eight statements on heat intolerance, heat sensation, cold intolerance, and cold sensation. Responses were recorded on a 5-point Likert scale ranging from 1 to 5. For heat intolerance, the following statements were included: “I usually have an aversion to heat” and “I usually prefer cool or cold.” The sum of the two responses was recorded as the level of heat intolerance (range: 2–10). Likewise, the following statements were included for heat sensation: “I usually have a warm sensation in the body or feel hot” and “I usually feel a hot or burning sensation in the body;” for cold intolerance, “I usually have an aversion to cold” and “I usually prefer warmth;” and for cold sensation, “My hands and feet are usually cold” and “I usually feel a cold sensation in the body.”

### Definition of MetS

MetS was defined according to the modified Third National Cholesterol Education Program/Adult Treatment Panel (NCEP-ATP III), whereas the WC cutoff points for central obesity were based on the Korean Society for the Study of Obesity [42, 43]. The presence of at least three of the following five criteria was defined as MetS: (1) waist circumference (WC) ≥ 85 cm; (2) triglyceride (TG) level ≥ 150 mg/dL or taking dyslipidemia medication; (3) high-density lipoprotein cholesterol level < 50 mg/dL; (4) systolic BP ≥ 130 mmHg, diastolic BP ≥ 85 mmHg, or taking antihypertensive medication; and (5) FBG level ≥ 100 mg/dL or taking antihyperglycemic medication.

### Lifestyle variables

Alcohol consumption categories were defined by average volume of alcohol consumption per day according to the World Health Organization report: non-drinker (0 g/day of alcohol), responsible drinking (0.1–19.99 g of pure alcohol daily), hazardous drinking (20–39.99 g of pure alcohol daily), and harmful drinking (≥ 40 g of pure alcohol daily) [44]. Smoking categories included currently smoking, smoked in the past, and non-smokers. Physical activity level was evaluated using the Korean Global Physical Activity Questionnaire [45] and classified into the following three categories based on the total physical activity levels per week: low, moderate, and high.

### Statistical analyses

Values are presented as mean ± standard deviation. The differences between individuals with or without MetS were assessed using Student’s *t*-test for normally distributed data or Mann–Whitney test for non-normally distributed data. To identify individuals with higher or lower thermal intolerance/sensation than expected based on their obesity levels, generalized additive models (GAMs) for each of the thermal intolerance/sensation were fitted using BMI and waist–hip ratio (WHR), whereas smooth functions were estimated using thin plate regression splines. GAMs provide a general framework for extending a standard linear model by allowing non-linear functions of each variable while maintaining additivity [46]. The optimal effective degrees of freedom were chosen automatically by means of generalized cross validation (GCV) [47]. Groups were determined based on the difference between the original and predicted values using the model (Diff) and its relation to the mean absolute difference (MAD). If Diff was higher than MAD (Diff > MAD), the individual was allocated to the H group (higher than predicted group). If Diff was lower than MAD (Diff < MAD), the individual was allocated to the L group (lower than predicted group). If the absolute value of Diff was less than or equal to MAD (|Diff|≤ MAD), the individual was allocated to the R group (reference group). Multiple logistic regression models were then used to investigate the association between the groups and incidence of MetS, after adjusting for age, BMI, alcohol consumption, smoking status, and physical activity level.

The number of conditions of thermal intolerance/sensation that were related to the increased incidence of MetS was counted for each individual. The differences between groups based on the number of conditions were assessed using analysis of variance for normally distributed data or Kruskal–Wallis test for non-normally distributed data. Multiple logistic regression models were used to investigate the association between the number of conditions and incidence of MetS, after adjusting for the aforementioned confounders. *P* for trend was calculated using a multiple logistic regression model with adjustments for the number of conditions for continuous variables. All statistical analyses were performed using the R software version 4.0.5 (R Foundation for Statistical Computing, Vienna, Austria). GAMs were fitted using the MGCV package version 1.8–34.

## Results

Among 849 participants, 81 (9.5%) had MetS. Individuals with MetS were older and had higher BMI, WC, WHR, body fat, heat intolerance, heat sensation, and lower cold intolerance and sensation than those without MetS (Table 1).

**Table 1:** Characteristics of study participants (N = 849)

	No MetS	MetS	*P*
	(n = 768)	(n = 81)	
Age, years	42.0 ± 5.9	44.4 ± 5.6	0.001
BMI, kg/m^2^	23.0 ± 3.2	28.2 ± 4.2	< 0.001
WC, cm	78.4 ± 8.0	91.1 ± 9.9	< 0.001
WHR	0.8 ± 0.1	0.9 ± 0.1	< 0.001
Body fat, %	32.9 ± 5.6	39.2 ± 5.5	< 0.001
SBP, mmHg	111.9 ± 13.6	129.5 ± 16.5	< 0.001
DBP, mmHg	69.1 ± 10.4	80.7 ± 13.0	< 0.001
FBG, mg/dL	80.8 ± 12.6	94.9 ± 25.0	< 0.001
TG, mg/dL	94.4 ± 52.0	181.4 ± 80.3	< 0.001
HDL-C, mg/dL	61.8 ± 13.6	46.9 ± 9.9	< 0.001
Heat intolerance	5.5 ± 1.9	6.8 ± 2.2	< 0.001
Heat sensation	4.8 ± 2.0	6.4 ± 2.1	< 0.001
Cold intolerance	7.6 ± 2.0	6.1 ± 2.2	< 0.001
Cold sensation	6.3 ± 2.2	5.4 ± 2.3	< 0.001

Data are presented as mean ± standard deviation

*MetS*, metabolic syndrome; *BMI*, body mass index; *WC*, waist circumference; *WHR*, waist–hip ratio; *SBP*, systolic blood pressure; *DBP*, diastolic blood pressure; *FBG*, fasting blood glucose; *TG*, triglyceride; *HDL-C*, high-density lipoprotein cholesterol

**Fig. 1:**
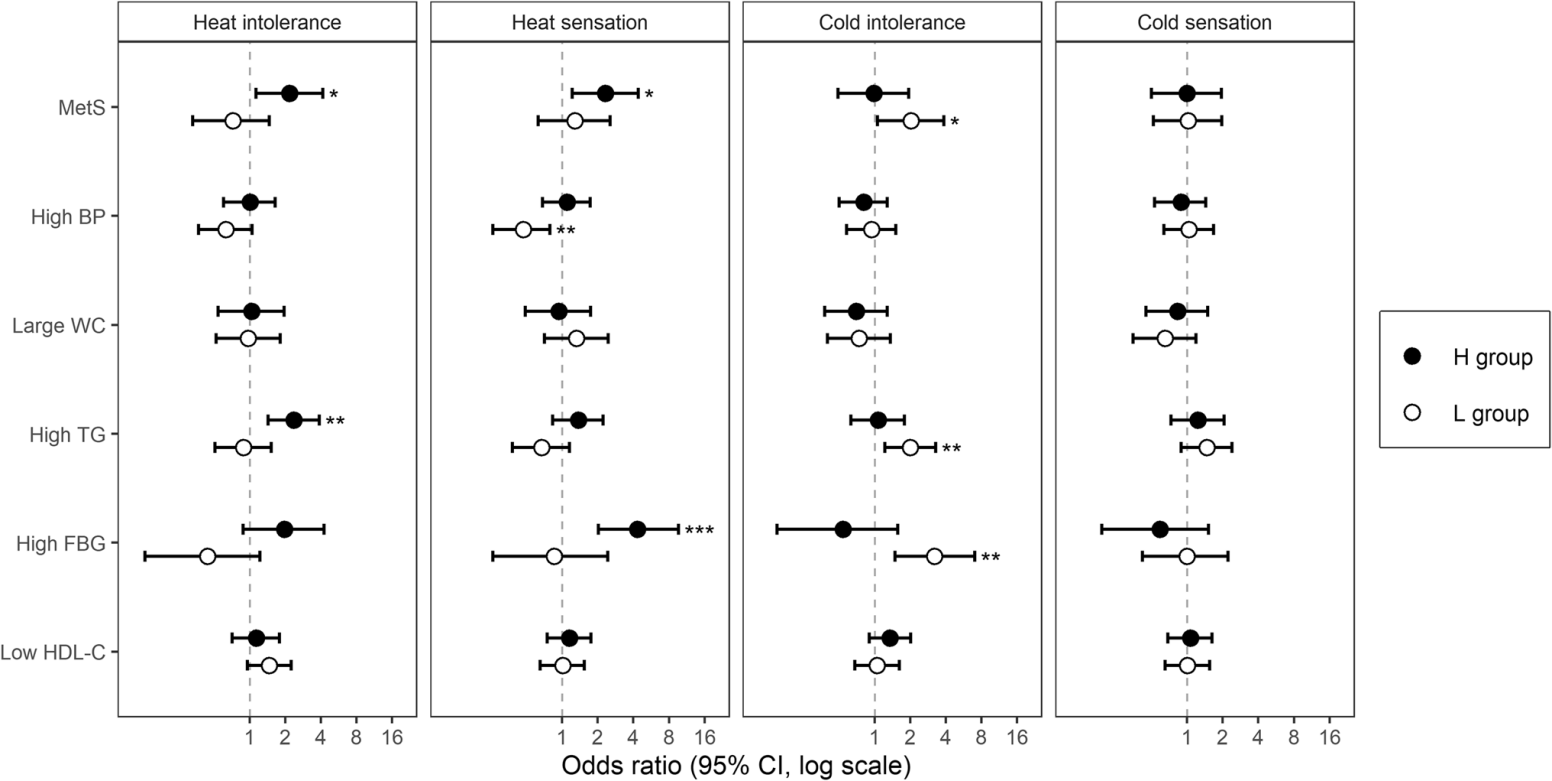
Odds ratios with 95% CI for the association of higher or lower thermal intolerance/sensation with MetS and its components. Multiple logistic regression analysis was used to calculate the odds ratio with reference to the R group (reference group). The model was adjusted for age, BMI, alcohol consumption, smoking status, and physical activity level. CI, confidence interval; MetS, metabolic syndrome; BP, blood pressure; WC, waist circumference; TG, triglyceride; FBG, fasting blood glucose; HDL-C, high-density lipoprotein cholesterol; H, higher than predicted group; L, lower than predicted group; ^*^, *P* < 0.05; ^**^, *P* < 0.01; ^***^, *P* < 0.001

Regarding heat intolerance, the H group had a higher likelihood of having MetS (odds ratio [OR]: 2.18 [95% confidence interval, 95% CI: 1.13–4.15]) and high TG levels (OR: 2.36 [95% CI: 1.43–3.89]) than the R group. Regarding heat sensation, the H group had a higher likelihood of having MetS (OR: 2.32 [95% CI: 1.22–4.40]) and high FBG levels (OR: 4.34 [95% CI: 2.02–9.63]) than the R group, whereas the L group had a lower likelihood of having high BP (OR: 0.47 [95% CI: 0.26–0.79]) than the R group. Regarding cold intolerance, the L group had a higher likelihood of having MetS (OR: 2.03 [95% CI: 1.06–3.86]), high TG levels (OR: 2.01 [95% CI: 1.22–3.29]), and high FBG levels (OR: 3.20 [95% CI: 1.49–7.00]) than the R group. Regarding cold sensation, the H and L groups did not have a significantly different likelihood of having MetS or its components (Fig. 1) compared with the R group.

As higher heat intolerance, higher heat sensation, and lower cold intolerance were significantly associated with the prevalence of MetS and its components, the number of these conditions was accordingly counted for each participant. The characteristics of the participants according to the number of conditions are shown in Table 2. There were no significant differences in age, BMI, WC, WHR, or body fat; however, the prevalence of MetS and the level of HOMA-IR showed significant differences among the groups (Table 2).

**Table 2:** Characteristics of patients stratified by the number of conditions of higher heat intolerance, higher heat sensation, and lower cold intolerance

	None	One	Two	Three	*P*
	(n = 516)	(n = 189)	(n = 87)	(n = 57)	
Age, years	42.4 ± 5.8	41.9 ± 5.9	41.8 ± 6.2	42.8 ± 6.4	0.693
BMI, kg/m^2^	23.5 ± 3.7	23.2 ± 3.5	23.9 ± 3.7	23.6 ± 3.0	0.382
WC, cm	79.9 ± 9.3	78.7 ± 8.2	80.1 ± 9.3	79.9 ± 7.9	0.591
WHR	0.8 ± 0.1	0.8 ± 0.1	0.8 ± 0.1	0.8 ± 0.1	0.546
Body fat, %	33.6 ± 6.0	33.1 ± 5.3	33.5 ± 6.6	33.8 ± 5.4	0.596
MetS, *n* (%)	42 (8.1%)	16 (8.5%)	13 (14.9%)	10 (17.5%)	0.035
SBP, mmHg	113.3 ± 15.1	112.9 ± 14.4	115.4 ± 15.2	114.6 ± 13.8	0.275
DBP, mmHg	69.8 ± 11.5	70.1 ± 10.6	72.1 ± 11.7	71.3 ± 9.3	0.129
FBG, mg/dL	81.0 ± 10.1	81.4 ± 10.4	83.3 ± 10.1	92.4 ± 41.8	0.087
TG, mg/dL	100.1 ± 58.7	101.8 ± 52.0	106.1 ± 62.5	124.0 ± 94.6	0.308
HDL-C, mg/dL	60.1 ± 13.6	60.8 ± 15.0	60.8 ± 13.5	61.1 ± 14.7	0.871
LDL-C, mg/dL	114.3 ± 30.7	115.8 ± 32.9	113.0 ± 31.2	122.2 ± 38.4	0.442
HOMA-IR	1.2 ± 0.9	1.1 ± 0.6	1.4 ± 1.0	1.6 ± 1.8	0.038
Insulin, mIU/L	5.7 ± 4.2	5.2 ± 2.9	6.5 ± 4.4	6.4 ± 4.5	0.104
HbA1c, %	5.4 ± 0.4	5.4 ± 0.5	5.4 ± 0.4	5.7 ± 1.4	0.529
hs-CRP, mg/L	1.3 ± 3.0	1.3 ± 4.6	1.6 ± 3.9	1.8 ± 4.7	0.408
T3, ng/dL	104.3 ± 20.7	103.7 ± 22.6	111.6 ± 33.2	108.3 ± 26.6	0.385
T4, µg/dL	8.0 ± 1.4	7.9 ± 1.4	8.2 ± 1.6	8.3 ± 1.6	0.182
TSH, uIU/mL	1.9 ± 1.5	1.9 ± 1.3	1.7 ± 1.1	1.5 ± 0.9	0.082

Data are presented as mean ± standard deviation or numbers (%). *BMI*, body mass index; *WC*, waist circumference; *WHR,* waist–hip ratio; *MetS*, metabolic syndrome; *SBP*, systolic blood pressure; *DBP*, diastolic blood pressure; *FBG*, fasting blood glucose; *TG*, triglyceride; *HDL-C*, high-density lipoprotein cholesterol; *LDL-C*, low-density lipoprotein cholesterol; *HOMA-IR*, homeostasis model assessment of insulin resistance; *HbA1c*, hemoglobin A1c; *hs-CRP*, high-sensitivity C-reactive protein; TSH, thyroid stimulating hormone

**Fig. 2:**
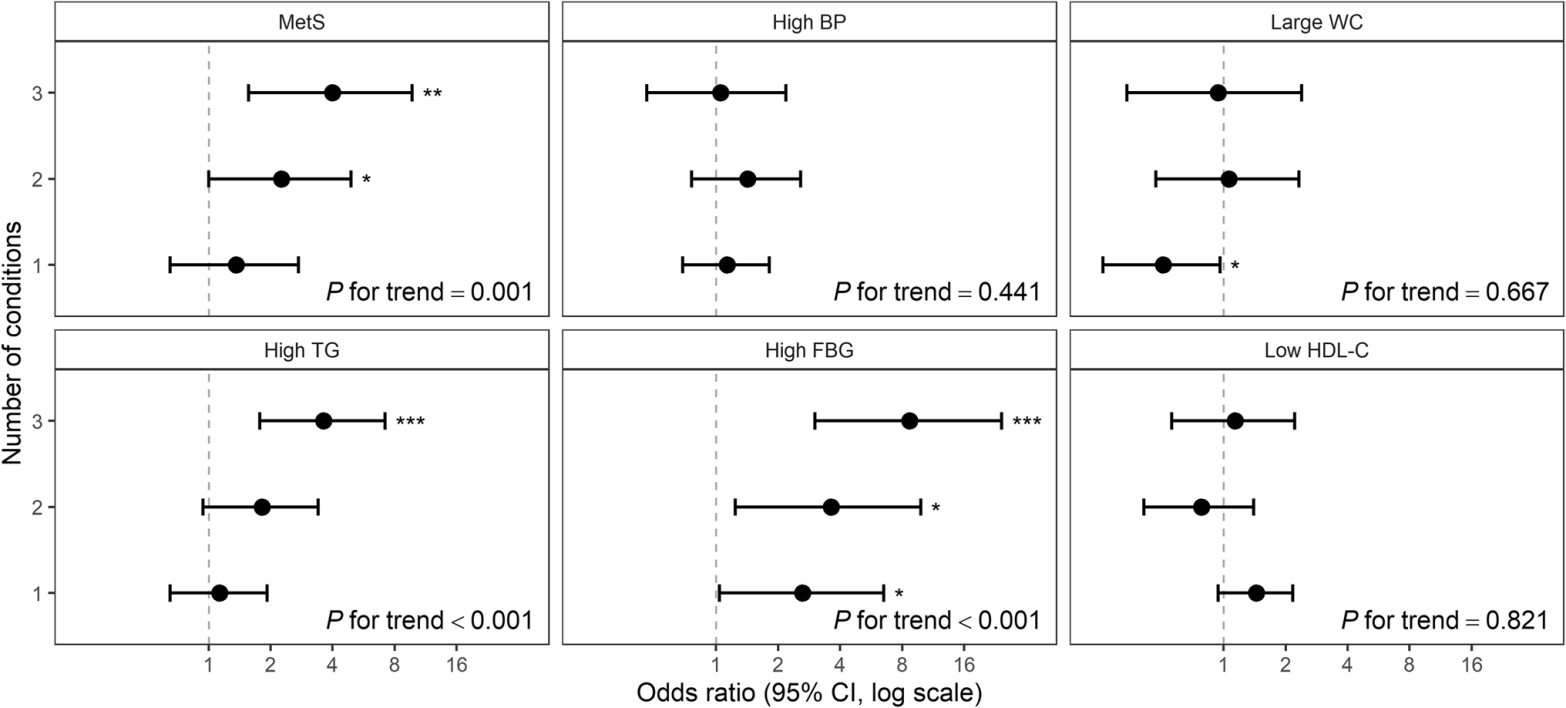
Odds ratios with 95% CI for the association of the number of conditions of thermal intolerance/sensation with MetS and its components. The counted conditions included higher heat intolerance, higher heat sensation, and lower cold intolerance. A multiple logistic regression analysis was used to calculate the odds ratio with reference to the group that has none of the conditions, adjusted for age, body mass index, alcohol consumption, smoking status, and physical activity level. *P* for trend was calculated using a multiple logistic regression model with adjustments for the number of conditions for continuous variables. CI, confidence interval; MetS, metabolic syndrome; BP, blood pressure; WC, waist circumference; TG, triglyceride; FBG, fasting blood glucose; HDL-C, high-density lipoprotein cholesterol; ^*^, *P* < 0.05; ^**^, *P* < 0.01; ^***^, *P* < 0.001

According to multiple logistic regression analysis results, the likelihood of having MetS was increased in those who had two (OR: 2.26 [95% CI: 1.00–4.92]) or three (OR: 4.00 [95% CI: 1.56–9.74]) conditions compared with those who had none of the conditions, and the linear trend was significant (*P* = 0.001). Regarding the components of MetS, the likelihood of having high FBG level was increased in those who had one (OR: 2.63 [95% CI: 1.04–6.52]), two (OR: 3.61 [95% CI: 1.24–9.84]), or three (OR: 8.69 [95% CI: 3.02–24.3]) conditions, and the linear trend was significant (*P* < 0.001). The likelihood of having high TG level was increased only in those who had three conditions (OR: 3.61 [95% CI: 1.77–7.19], and the linear trend was significant (*P* < 0.001) (Fig. 2).

## Discussion

This study aimed to investigate the association between self-identified thermal intolerance/sensation and MetS in order to verify the working hypothesis that individuals with altered thermal sensitivity may have a predisposition to MetS and its components. Our findings suggest that individuals with higher heat intolerance, lower cold intolerance, and higher heat sensation than expected had a higher likelihood of having MetS. Significant associations between thermal sensitivity and high glucose, triglyceride, and blood pressure levels were observed. These results support the working hypothesis, suggesting the potential clinical utility of evaluating thermal sensitivity as a PPPM approach for MetS.

We evaluated self-identified heat or cold intolerance based on participants’ subjective aversion to or preference for cold or heat; thus, they are related to the concept of thermal comfort. The thermal comfort zone is defined as the range of ambient temperatures within which an individual expresses satisfaction with the thermal environment, whereas the thermoneutral zone is defined as the range of ambient temperatures without regulatory changes in metabolic heat production or evaporative heat loss [48]. The relationship between an individual’s thermal comfort zone and the thermal neutral zone remains unclear; however, previous studies have reported that they might share similar ranges of temperatures [35, 49]. Having a higher heat intolerance and a lower cold intolerance might indicate a lower location of the thermal comfort zone or the thermoneutral zone. Recent studies have reported that cold exposure below the lower margin of the thermoneutral zone (lower critical temperature, LCT) increases thermogenesis and affects insulin sensitivity [8, 50]. In those in whom LCT is lower than that in others, a relatively colder environment is needed to elicit CIT, and a relatively mild cold exposure (above their LCT and below others’ LCT) might not cause thermogenesis, whereas it causes thermogenesis in others. Thus, those with a lower LCT might have reduced CIT and subsequently experience reduced beneficial effects of CIT on glucose metabolism. Moreover, those with a lower LCT might exhibit reduced cold acclimatization even in the same climate, which subsequently contributes to relatively reduced BAT activity.

Individuals with a higher heat sensation in the body had a higher prevalence of MetS and high FBG levels. Because thermal sensation and thermal intolerance are closely related [51], this could be explained by the above-mentioned association between thermal intolerance and MetS. However, it is also plausible that increased heat sensation might also be a sign of peripheral neuropathy, as multiple studies have demonstrated the association between neuropathy and MetS, as well as type II diabetes [52].

We found that a higher or lower cold sensation was neither significantly associated with MetS nor its components. However, previous studies have suggested that increased cold sensation in the extremities or Flammer syndrome reduces the risk of developing MetS or related diseases, including diabetes mellitus and dyslipidemia [18, 23, 24, 53]. The contradictory results might be attributed to the difference in the definitions of the conditions. We identified individuals with higher cold sensation based on their relatively higher cold sensation in the whole body compared with those with similar levels of obesity, whereas previous studies focused on the symptoms of cold sensation in the extremities irrespective of their obesity levels.

According to the NCEP-ATP III definition of MetS, there are 16 different combinations of components for diagnosing MetS, and the risk of future cardiovascular diseases and diabetes has been shown to differ depending on what combination of components are present [54, 55]. This means that significant heterogeneity exists among MetS patients, and patient stratification may be useful for a tailored approach toward patients with MetS. In this study, levels of FBG, TG, and BP were significantly associated with thermal intolerance/sensation, while WC and HDL were not. Thus, thermal sensitivity might have a stronger association with specific component combinations of MetS that are related to high levels of FBG, TG, and BP. This needs to be further clarified in future research with larger sample sizes.

MetS is generally discussed together with obesity because the prevalence of MetS is higher in obese than in non-obese individuals, and obesity is a powerful predictor of the key components of MetS [32, 33]; however, obesity is not always synonymous with MetS. So-called ‘metabolically healthy’ obese individuals constitute approximately 30% of overall obese individuals, and normal-weight individuals with metabolic abnormalities constitute approximately 20% of the normal-weight population [56]. It is also reported that underweight is related to more severe and extensive cardiovascular diseases that are related to MetS [34]. Therefore, MetS in non-obese individuals should not be overlooked, and risk factors for MetS that are independent of obesity should be investigated.

This study presents the potential utility of thermal sensitivity as a predictive measure for MetS, independently of obesity. Although thermal sensitivity is significantly associated with obesity level [28, 57], we have minimized the confounding effect of obesity through our approach to defining the groups and the statistical adjustments performed as part of the logistic regression. In detail, we defined higher or lower thermal intolerance/sensation based on the differences between the original values and those predicted by obesity levels. Thus, comparing the thermal intolerance/sensation levels of individuals with similar levels of obesity was possible. The differences in obesity levels among the R, L, and H groups were remarkably smaller based on our definition than when the original values of thermal intolerance/sensation were solely used (Online Resource 1). In addition, adjusting for BMI in the logistic regression models helped ensure that the significant association noted between thermal intolerance/sensation and MetS was not attributable to the confounding effect of obesity levels. Moreover, the resulting association was stronger than when the original values of thermal intolerance/sensation were solely used (Online Resources 2, 3, 4). Thus, our novel way of defining higher or lower thermal intolerance/sensation in consideration of obesity levels is a good way of investigating the independent association of thermal intolerance/sensation with MetS and useful in identifying participants at high risk for MetS.

This study had some limitations. Firstly, owing to the cross-sectional design, causality between thermal intolerance/sensation and MetS cannot be inferred from our findings. Secondly, because we only analyzed the data of women who were aged 30–55 years, the generalization of the results to men or other age groups is limited. Thirdly, subjective thermal intolerance/sensation may not accurately reflect the range and position of the thermoneutral zone. Although, subjective thermal intolerance/sensation was associated with the objective responses of the body to ambient temperature [37, 38], we could not ignore the effect of the person’s usual thermal environment, thermal behavior, and psychological aspects on their subjective thermal perception. To determine whether the differences in the position of the thermoneutral zone affected the prevalence of MetS, experimental studies evaluating the thermoneutral zone of each individual and its association with MetS should be conducted in the future. Lastly, the climate of the study participants’ residence may have affected the results. Daejeon City, the study setting, has a continental climate with four distinct seasons and a wide temperature difference between summer and winter, with an average annual temperature range of 26.6 °C [58]. Studies on participants living in areas with different seasonal changes or smaller annual temperature ranges might yield different results.

## Conclusions and expert recommendations

This study revealed the significant association between self-identified thermal intolerance/sensation and MetS and its components even at a similar obesity level. Individuals with higher heat intolerance, lower cold intolerance, and higher heat sensation than expected had a higher likelihood of having MetS.

### Identification of individuals at high risk of having MetS

From the perspectives of PPPM, the identification of individuals at high risk of having MetS is essential [59], so as to efficiently provide preventive measures to those predisposed to MetS and its related diseases. Many risk factors related to MetS are modifiable and therefore are preventable through lifestyle changes [3]. As our results indicate that individual differences in thermal sensitivity play an important role in identifying a high-risk group of MetS, it is recommended that the thermal sensitivity of each individual is recorded and included in the individualized patient profile for MetS, which helps to apply efficiently tailored strategies in individuals susceptible to MetS. As the association of thermal sensitivity with MetS was independent of obesity levels, thermal sensitivity evaluations may help identify non-obese individuals at risk of developing MetS, who have been all too commonly overlooked with regard to metabolic diseases [34]. Although this study focused on MetS, a wider scope of prevalent diseases linked to MetS, such as type II diabetes and cardiovascular diseases, might have similar associations with thermal sensitivity, which needs to be further investigated in future studies along with efforts to investigate the underlying pathomechanisms.

### Stratification of patients for the development of personalized protocols for CIT on metabolic disorders

In the recent years, CIT has gained considerable interest and has been proposed as a potential treatment for metabolic disorders, as it leads to an increase in energy expenditure, oxidation of glucose and triglycerides as substrates, and insulin sensitivity enhancement [50, 60, 61]; Although lifestyle programs involving exercise or diet are effective for metabolic disorders, long-term adherence is often not realized, justifying the need for other approaches, such as environmental adjustment (i.e., cold exposure) [8]. However, the duration, timing, and temperatures for CIT that are most effective to induce an increase in thermogenesis and are thus treat metabolic diseases have not yet been determined [62]. We speculate that the conditions vary largely between individuals owing to the variability in thermal sensitivity among individuals. For example, individuals who are more heat-sensitive and less cold-sensitive (i.e. those having higher heat intolerance, higher sensation in body, and lower cold intolerance) than others might need a lower temperature or longer duration of cold exposure to elicit a sufficient amount of CIT to prevent or treat MetS. As higher heat intolerance/sensation and lower cold intolerance were significantly related to only three components of MetS in this study, we speculate that this association might present only in some of the component combination groups of MetS. Thus, advanced patient stratification based on various factors including thermal sensitivity and component combinations is needed to develop personalized protocols for CIT.

### Importance of considering obesity levels in thermal sensitivity research

The symptoms related to thermal sensitivity, such as feeling inappropriately cold, and cold hypersensitivity in hands/feet have recently been vigorously studied with regard to risk assessment and patient stratification in diseases such as ocular diseases, breast cancer, and multiple sclerosis [18, 19]. As one of the factors that considerably influence thermal sensitivity, obesity level needs be considered to evaluate an individual’s thermal sensitivity as normal or abnormal. Several physiologic changes that accompany obesity tend to increase heat production (increased resting metabolic heat production due to the large fat-free mass) or impede heat loss (increased thermal insulation due to a large volume of adipose tissue and a lower ratio of surface area to body mass). Accordingly, obese people tend to feel hotter and have a higher skin temperature in their hands than non-obese people under the same environmental conditions [25, 26]. As one of the practical ways to consider obesity level in the evaluation of thermal sensitivity, we suggest comparing thermal sensitivity levels between individuals with a similar level of obesity. This could help to investigate the independent effect of thermal sensitivity from obesity levels, and facilitate the incorporation of thermal sensitivity in the PPPM approach toward various diseases.

## Supplementary Information

Below is the link to the electronic supplementary material.

Supplementary file1 (PDF 158 KB)

Supplementary file2 (PDF 219 KB)

Supplementary file3 (PDF 184 KB)

Supplementary file4 (PDF 258 KB)

## Data Availability

The data used to support the findings of this study were supplied by the Korean Medicine Data Center of the Korea Institute of Oriental Medicine under license; thus, they cannot be made freely available. Requests for access to these data should be made to the Korean Medicine Data Center (http://kdc.kiom.re.kr/html/).
