# Supplementary material for: Evaluation of thermal sensitivity is of potential clinical utility for the predictive, preventive, and personalized approach advancing metabolic syndrome management
Source: EPMA J. 2022 Feb 18;13(1):125–35. doi: 10.1007/s13167-022-00273-6 (PMC8897525; doi:10.1007/s13167-022-00273-6)
Supplement: Supplementary file 1 — Supplementary file1 (PDF 158 KB) [file 13167_2022_273_MOESM1_ESM.pdf]

Evaluation of thermal sensitivity is of potential clinical utility for the predictive, preventive, and personalized approach advancing metabolic syndrome management

EPMA Journal

Sujeong Mun, Kihyun Park, Siwoo Lee

KM Data Division, Korea Institute of Oriental Medicine, Daejeon, Republic of Korea

***Corresponding Author**

Siwoo Lee

ifree72@gmail.com

Online Resource 1. Comparison of obesity levels between groups stratified according to thermal intolerance/sensation

		Groups according to relative values of thermal intolerance/sensation compared with those predicted by the model				Groups according to quartiles of thermal intolerance/sensation			
		R	L	H	*P*	*Q2 & Q3*	*Q1*	*Q4*	*P*
Heat intolerance	BMI, kg/m^2	23.3 ± 3.5	23.8 ± 3.8	23.7 ± 3.8	0.172	23.5 ± 3.4	22.3 ± 2.6	26.0 ± 4.6	<0.001
	WC, cm	79.3 ± 8.9	80.7 ± 9.4	79.7 ± 8.9	0.124	79.5 ± 8.6	77.0 ± 7.1	85.0 ± 11.1	<0.001
	WHR	0.8 ± 0.1	0.8 ± 0.1	0.8 ± 0.1	0.156	0.8 ± 0.1	0.8 ± 0.1	0.9 ± 0.1	<0.001
	Body fat, %	33.3 ± 5.9	34.1 ± 5.9	33.6 ± 6.0	0.310	33.5 ± 5.8	31.9 ± 5.3	36.6 ± 6.4	<0.001
Heat intolerance	BMI, kg/m^2	23.7 ± 3.7	23.2 ± 3.6	23.4 ± 3.4	0.048	23.6 ± 3.4	22.4 ± 2.9	25.6 ± 4.3	<0.001
	WC, cm	79.8 ± 9.1	79.4 ± 9.1	79.4 ± 8.6	0.761	79.7 ± 8.6	77.2 ± 7.9	84.4 ± 9.8	<0.001
	WHR	0.8 ± 0.1	0.8 ± 0.1	0.8 ± 0.1	0.917	0.8 ± 0.1	0.8 ± 0.1	0.9 ± 0.1	<0.001
	Body fat, %	33.9 ± 5.8	32.7 ± 6.2	33.2 ± 5.8	0.024	33.7 ± 5.8	32.0 ± 5.6	36.1 ± 5.8	<0.001
Cold intolerance	BMI, kg/m^2	23.6 ± 4.0	23.6 ± 3.1	23.2 ± 3.2	0.244	23.1 ± 3.1	24.8 ± 4.1	22.3 ± 2.9	<0.001
	WC, cm	79.9 ± 9.8	79.5 ± 8.0	79.1 ± 8.1	0.576	78.8 ± 8.5	82.6 ± 9.5	76.7 ± 7.7	<0.001
	WHR	0.8 ± 0.1	0.8 ± 0.1	0.8 ± 0.1	0.591	0.8 ± 0.1	0.9 ± 0.1	0.8 ± 0.1	<0.001
	Body fat, %	33.6 ± 6.4	33.5 ± 5.5	33.3 ± 5.3	0.614	33.0 ± 5.8	35.1 ± 6.0	31.9 ± 5.4	<0.001
Cold sensation	BMI, kg/m^2	23.4 ± 3.8	23.9 ± 3.4	23.4 ± 3.2	0.048	22.9 ± 3.0	24.9 ± 4.1	22.3 ± 3.0	<0.001
	WC, cm	79.6 ± 9.2	79.9 ± 8.9	79.4 ± 8.4	0.657	78.4 ± 8.1	82.6 ± 9.7	76.5 ± 8.2	<0.001
	WHR	0.8 ± 0.1	0.8 ± 0.1	0.8 ± 0.1	0.635	0.8 ± 0.1	0.9 ± 0.1	0.8 ± 0.1	<0.001
	Body fat, %	33.2 ± 6.1	33.9 ± 5.6	33.8 ± 5.5	0.081	32.7 ± 5.7	35.2 ± 5.9	32.0 ± 5.6	<0.001

Data are presented as mean ± standard deviation.

R, reference group; L, lower than predicted group; H, higher than predicted group; BMI, body mass index; WC, waist circumference; WHR, waist–hip ratio
