# Supplementary material for: Evaluation of thermal sensitivity is of potential clinical utility for the predictive, preventive, and personalized approach advancing metabolic syndrome management
Source: EPMA J. 2022 Feb 18;13(1):125–35. doi: 10.1007/s13167-022-00273-6 (PMC8897525; doi:10.1007/s13167-022-00273-6)
Supplement: Supplementary file 2 — Supplementary file2 (PDF 219 KB) [file 13167_2022_273_MOESM2_ESM.pdf]

# Evaluation of thermal sensitivity is of potential clinical utility for the predictive, preventive, and personalized approach advancing metabolic syndrome management

*EPMA Journal*

Sujeong Mun, Kihyun Park, Siwoo Lee

KM Data Division, Korea Institute of Oriental Medicine, Daejeon, Republic of Korea

***Corresponding Author**

Siwoo Lee

ifree72@gmail.com

**Online Resource 2.** Odds ratios with 95% CI for the association of the first and fourth quartile groups of thermal intolerance/sensation with MetS and its components

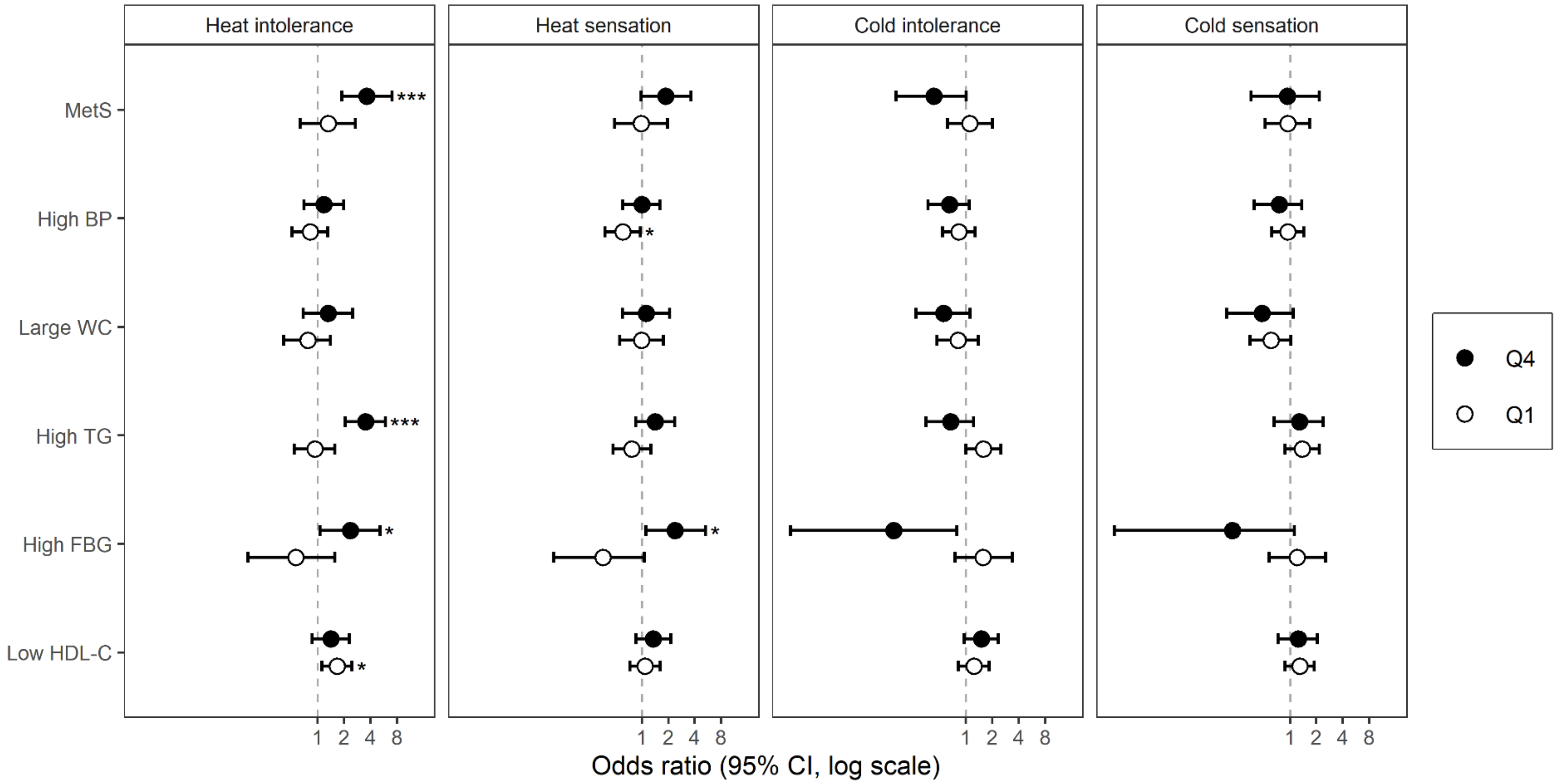


Multiple logistic regression analysis was used to calculate the odds ratio with reference to the Q2 and Q3 groups. The model was adjusted for age, BMI, alcohol consumption, smoking status, and physical activity level.

CI, confidence interval; MetS, metabolic syndrome; BP, blood pressure; WC, waist circumference; TG, triglyceride; FBG, fasting blood glucose; HDL-C, high-density lipoprotein cholesterol; *, $P < 0.05$; **, $P < 0.01$; ***, $P < 0.001$
