# Supplementary material for: Evaluation of thermal sensitivity is of potential clinical utility for the predictive, preventive, and personalized approach advancing metabolic syndrome management
Source: EPMA J. 2022 Feb 18;13(1):125–35. doi: 10.1007/s13167-022-00273-6 (PMC8897525; doi:10.1007/s13167-022-00273-6)
Supplement: Supplementary file 3 — Supplementary file3 (PDF 184 KB) [file 13167_2022_273_MOESM3_ESM.pdf]

Evaluation of thermal sensitivity is of potential clinical utility for the predictive, preventive, and personalized approach advancing metabolic syndrome management

EPMA Journal

Sujeong Mun, Kihyun Park, Siwoo Lee

KM Data Division, Korea Institute of Oriental Medicine, Daejeon, Republic of Korea

***Corresponding Author**

Siwoo Lee

ifree72@gmail.com

Online Resource 3. Characteristics of participants based on the number of conditions of higher (Q4) heat intolerance, higher (Q4) heat sensation, and lower (Q1) cold intolerance

	None (n=516)	One (n=189)	Two (n=87)	Three (n=57)	*P*
Age, years	42.0 ± 5.7	43.1 ± 5.9	42.5 ± 5.7	41.4 ± 7.1	0.039
BMI, kg/m^2	22.5 ± 2.8	24.0 ± 3.4	25.2 ± 4.4	26.7 ± 4.6	<0.001
WC, cm	77.3 ± 7.7	80.9 ± 8.5	83.4 ± 10.5	86.6 ± 10.3	<0.001
WHR	0.8 ± 0.1	0.8 ± 0.1	0.9 ± 0.1	0.9 ± 0.1	<0.001
Body fat, %	32.2 ± 5.6	34.1 ± 5.5	35.8 ± 6.1	37.3 ± 6.0	<0.001
MetS, n(%)	22 (4.6%)	22 (10.7%)	16 (16.3%)	21 (30.4%)	<0.001
SBP, mmHg	111.2 ± 14.0	115.8 ± 15.6	115.7 ± 14.8	119.8 ± 15.2	<0.001
DBP, mmHg	68.5 ± 10.6	72.1 ± 11.8	72.0 ± 11.2	74.1 ± 10.9	<0.001
FBG, mg/dL	79.8 ± 7.4	83.2 ± 14.2	83.5 ± 9.1	92.8 ± 38.4	<0.001
TG, mg/dL	93.9 ± 55.1	100.5 ± 44.7	120.1 ± 71.5	145.1 ± 94.4	<0.001
HDL-C, mg/dL	61.7 ± 13.8	59.8 ± 13.4	58.5 ± 15.2	56.1 ± 14.3	0.001
LDL-C, mg/dL	112.4 ± 30.5	118.6 ± 31.4	113.4 ± 33.1	125.4 ± 37.1	0.002
HOMA-IR	1.0 ± 0.7	1.2 ± 0.7	1.4 ± 0.8	2.2 ± 2.1	<0.001
Insulin, mIU/L	5.1 ± 3.0	5.7 ± 3.3	6.4 ± 3.5	9.2 ± 8.0	<0.001
HbA1c, %	5.3 ± 0.3	5.5 ± 0.6	5.4 ± 0.4	5.9 ± 1.3	<0.001
hs-CRP, mg/L	1.2 ± 3.9	1.2 ± 2.3	1.4 ± 3.4	2.4 ± 4.7	<0.001
T3, ng/dL	103.9 ± 22.9	104.0 ± 22.0	109.1 ± 26.9	111.5 ± 21.7	0.022
T4, ug/dL	8.0 ± 1.4	8.0 ± 1.5	8.2 ± 1.6	8.3 ± 1.2	0.219
TSH, uIU/mL	1.8 ± 1.4	1.9 ± 1.6	1.7 ± 1.2	1.8 ± 1.1	0.828

Data are presented as mean ± standard deviation or *n* (%). BMI, body mass index; WC, waist circumference; WHR, waist-hip ratio; MetS, metabolic syndrome; SBP, systolic blood pressure; DBP, diastolic blood pressure; FBG, fasting blood glucose; TG, triglyceride; HDL-C, high-density lipoprotein cholesterol; LDL-C, low-density lipoprotein cholesterol; HOMA-IR, homeostasis model assessment of insulin resistance; HbA1c, hemoglobin A1c; hs-CRP, high-sensitivity C-reactive protein; TSH, thyroid stimulating hormone
