# Supplementary material for: Evaluation of thermal sensitivity is of potential clinical utility for the predictive, preventive, and personalized approach advancing metabolic syndrome management
Source: EPMA J. 2022 Feb 18;13(1):125–35. doi: 10.1007/s13167-022-00273-6 (PMC8897525; doi:10.1007/s13167-022-00273-6)
Supplement: Supplementary file 4 — Supplementary file4 (PDF 258 KB) [file 13167_2022_273_MOESM4_ESM.pdf]

Evaluation of thermal sensitivity is of potential clinical utility for the predictive, preventive, and personalized approach advancing metabolic syndrome management

EPMA Journal

Sujeong Mun, Kihyun Park, Siwoo Lee

KM Data Division, Korea Institute of Oriental Medicine, Daejeon, Republic of Korea

***Corresponding Author**

Siwoo Lee

ifree72@gmail.com

Online Resource 4. Odds ratios with 95% CI for the association of the number of conditions of thermal intolerance/sensation with MetS and its components

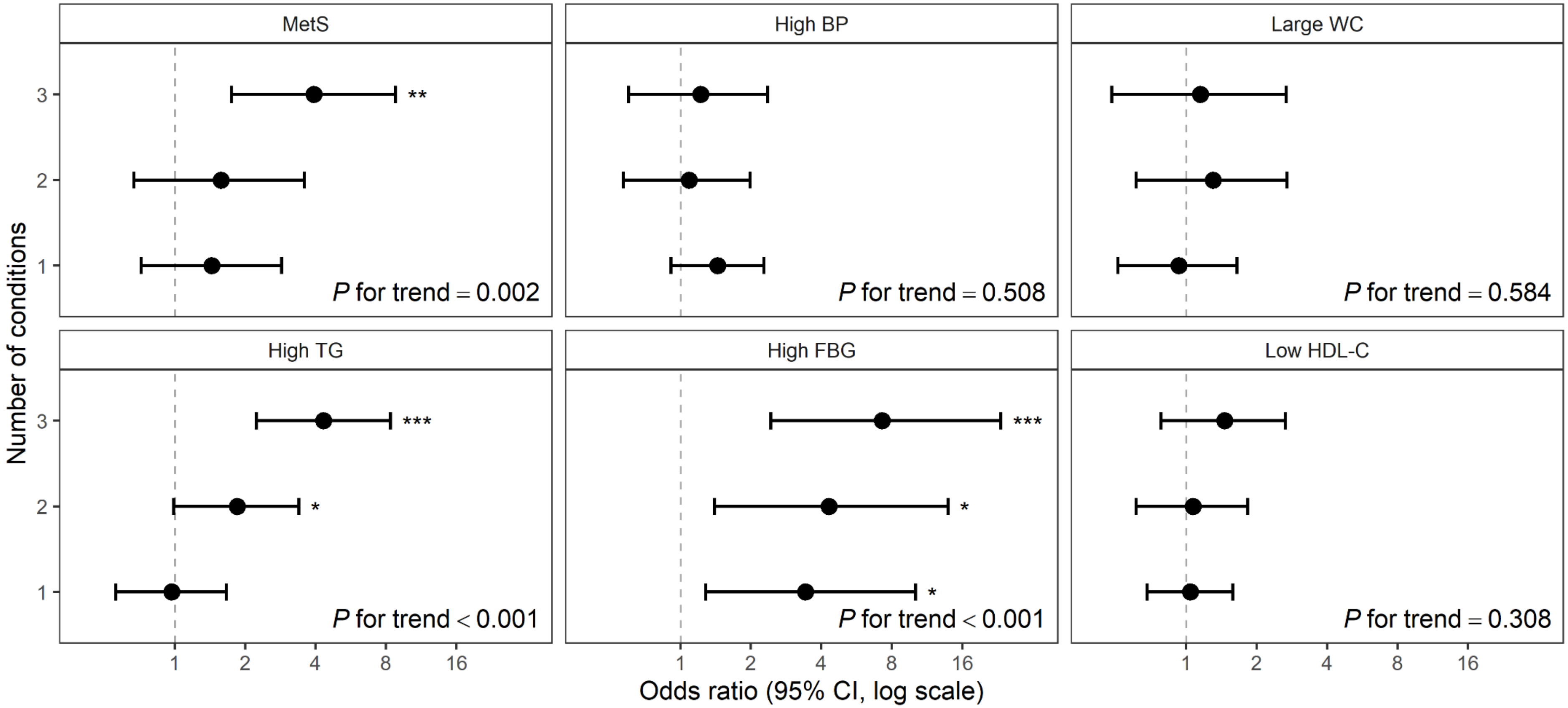

Higher/lower thermal intolerance/sensation was defined by the quartiles of the absolute value. The counted conditions included higher (Q4) heat intolerance, higher (Q4) heat sensation, and lower (Q1) cold intolerance. A multiple logistic regression analysis was used to calculate the odds ratio with reference to the group that has none of the conditions, adjusted for age, body mass index, alcohol consumption, smoking status, and physical activity level. *P* for trend was calculated using a multiple logistic regression model with adjustments for the number of conditions for continuous variables.

MetS, metabolic syndrome; BP, blood pressure; WC, waist circumference; TG, triglyceride; FBG, fasting blood glucose; HDL-C, high-density lipoprotein cholesterol; *, $P <$

0.05; **, $P < 0.01$; ***, $P < 0.001$
